# The bone morphogenetic protein receptor-1A pathway is required for lactogenic differentiation of mammary epithelial cells in vitro

**DOI:** 10.1007/s11626-012-9522-z

**Published:** 2012-06-23

**Authors:** C. Perotti, Ö. Karayazi, S. Moffat, C. S. Shemanko

**Affiliations:** Department of Biological Sciences, University of Calgary, Calgary, AB Canada T2N 1N4

**Keywords:** Bone morphogenetic protein receptor-1A, SMAD, Mammary epithelial cells, HC11 cells, Differentiation, Short interfering RNA, Prolactin, Noggin

## Abstract

Bone morphogenetic proteins (BMPs) have been implicated in the control of proliferation, tissue formation, and differentiation. BMPs regulate the biology of stem and progenitor cells and can promote cellular differentiation, depending on the cell type and context. Although the BMP pathway is known to be involved in early embryonic development of the mammary gland via mesenchymal cells, its role in later epithelial cellular differentiation has not been examined. The majority of the mammary gland development occurs post-natal, and its final functional differentiation is characterized by the emergence of alveolar cells that produce milk proteins. Here, we tested the hypothesis that bone morphogenetic protein receptor 1A (BMPR1A) function was required for mammary epithelial cell differentiation. We found that the BMPR1A-SMAD1/5/8 pathway was predominantly active in undifferentiated mammary epithelial cells, compared with differentiated cells. Reduction of BMPR1A mRNA and protein, using short hairpin RNA, resulted in a reduction of SMAD1/5/8 phosphorylation in undifferentiated cells, indicating an impact on this pathway. When the expression of the BMPR1A gene knocked down in undifferentiated cells, this also prevented beta-casein production during differentiation of the mammary epithelial cells by lactogenic hormone stimulation. Addition of Noggin, a BMP antagonist, also prevented beta-casein expression. Together, this demonstrated that BMP-BMPR1A-SMAD1/5/8 signal transduction is required for beta-casein production, a marker of alveolar cell differentiation. This evidence functionally identifies BMPR1A as a potential new regulator of mammary epithelial alveolar cell differentiation.

## Introduction

In addition to the roles of the bone morphogenetic protein (BMP) pathway controlling cell fate in embryonic stem cells, it has tissue-specific roles in epithelial stem cell maintenance and progenitor cell differentiation in the hair follicle (Kobielak et al. 2003; Guha et al. 2004; Zhang et al. 2006), prevents premature differentiation of myogenic satellite cells (Ono et al. 2011), and a role in adipocyte lineage determination from stem cells (Huang et al. 2009). Given these roles, we investigated its requirement for mammary epithelial cell differentiation.

BMPs bind to a complex of types 1 (bone morphogenetic protein receptor 1A (BMPR1A) and BMPR1B) and 2 (BMPR2) receptors, leading to phosphorylation of the type 1 receptors by the kinase domain of the type 2 receptors. Activation of the canonical pathway results in phosphorylation of specific SMAD proteins such as SMAD1, SMAD5 and SMAD8 (SMAD1/5/8). These complex with co-SMAD4 and translocate to the nucleus to affect gene regulation. Type 1 receptors dictate the specificity of ligand binding, and in the case of BMPR1A, it is BMP 2, BMP4, and to a lesser extent BMP7 (Botchkarev 2003).

The role of the BMP pathway is implicated in early mammary gland development. BMP4 is known to be important in ductal growth during embryonic mammary gland development, with the BMPR1A (also known as activin-like kinase 3) playing a role in the mammary mesenchyme, as opposed to the epithelium (Hens et al. 2007). BMP4 was also able to potentiate fibroblast growth factor (FGF)-2-, FGF-7-, FGF-10-, epidermal growth factor (EGF)-, or hepatocyte growth factor-induced cellular proliferation in J3B1A mammary epithelial cells, although BMP4 did not affect proliferation on its own (Montesano et al. 2008). In 3D collagen cultures of J3B1A cells, BMP4 disrupted spontaneous cellular morphology of polarized acinar structures in a pathway dependent on mitogen-activated protein kinases (Montesano 2007). The role of the BMP pathway in lactogenic mammary cell differentiation has not yet been examined.

It is believed that lactogenic mammary epithelial cells differentiate from luminal progenitors (Welm et al. 2002; Shackleton et al. 2006; Sleeman et al. 2006; Stingl et al. 2006; Asselin-Labat et al. 2007). HC11 cells, a prolactin responsive clone of the COMMA-1D mouse (Balb/c) mammary epithelial cell line, (Ball et al. 1988) have been used as an in vitro model to study mammary epithelial cell differentiation of the luminal lineage. HC11 cells are a well-known cell line that can be cultured to represent undifferentiated cells (with EGF and insulin), competent to differentiate (EGF), and differentiated (dexamethasone, insulin, and prolactin) cells. Competent cells (cultured in EGF) can be pre-differentiated with the lactogenic hormones dexamethasone and insulin prior to treatment with prolactin, which stimulates HC11 cells to produce markers of alveolar cell differentiation (Desrivieres et al. 2003; Perotti et al. 2009) including beta-casein. Previously, we identified *Bmpr1a* in a screen designed to identify potential regulators of differentiation, as a gene that was preferentially expressed in undifferentiated HC11 cells compared with differentiated HC11 cells (Perotti et al. 2009). We hypothesize that a reduction in BMPR1A signaling would disturb mammary epithelial progenitor cell differentiation. We now demonstrate that BMPR1A activity in HC11 mammary epithelial cells is required prior to lactogenic signal transduction, for the stage of milk-secretory cell differentiation marked by beta-casein production.

## Materials and Methods

### Antibodies.

Goat and rabbit anti BMPR1A antibodies were purchased from Santa Cruz (Santa Cruz, CA) and Abgent (Biolynx, San Diego, CA), respectively. Goat anti beta-casein (M-14) was obtained from Santa Cruz. Mouse anti-GRB2 and anti-phospho-SMAD1/5/8 (SMAD1,5 phospho-Ser 463/465, SMAD8 phospho-Ser 426/428) antibodies were purchased from Cell Signaling Technology (Pickering, ON, Canada), Histone H3 from Millipore (Billerica, MA). Secondary antibodies, HRP conjugated, were obtained from Bio-Rad (Mississauga, ON, Canada) and Pierce (Nepean, ON, Canada), fluorescence conjugated from Invitrogen (Burlington, ON, Canada).

### Materials.

RPMI 1640 medium was purchased from Hyclone (Nepean, ON, Canada). Glutamine, penicillin/streptomycin, and trypsin were from Gibco-Invitrogen. Insulin (bovine) and prolactin (ovine) were from Sigma (Oakville, ON, Canada), and EGF (mouse) from BD Biosciences (Mississauga, ON, Canada). The recombinant human Noggin/Fc chimera was purchased from R&D Systems (Minneapolis, MN).

### Cell culture.

Mouse HC11 cells (Ball et al. 1988) were grown in RPMI 1640 medium containing 10 % fetal calf serum, 2 mM l-glutamine and 100 units/ml penicillin–streptomycin. For cell maintenance, the medium was supplemented with insulin 5 μg/ml and EGF 0.01 μg/ml.

### Differentiation of HC11 cells.

For differentiation, 0.7 × 10^5^ HC11 undifferentiated cells/ml were cultured in 100 mm dishes during 4 d in RPMI media (+5 μg/ml insulin+10 μg/ml EGF). During this time cells reached 100 % confluence. Cells were then washed with PBS three times and media was replaced with competence media (+EGF) and were maintained for 1 d, after which they become responsive to lactogenic hormones. Then cells were washed and pre-differentiation media was added (+insulin + dexamethasone 10^−7^ M) for 1 d. Finally, cells were cultured for 3 d in differentiation media (+insulin + dexamethasone + prolactin 5 μg/ml) (Perotti et al. 2009). If included in the experiment, Noggin was added at the concentration indicated at the start of the differentiation procedure. Differentiation was monitored by measuring beta-casein production, using western blot and PCR.

### Protein extracts.

The cells taken at the appropriate time for undifferentiated, competent and differentiated stages were scraped with a rubber policeman in PBS on ice, centrifuged at 4°C and resuspended in NP40 lysis buffer, containing a set of protease inhibitors, 20 mM Tris-HCl, 150 mM NaCl, 2 mM EDTA, 0.1 % Triton-X, and 1 % SDS (Perotti et al. 2009). From the supernatant, 50 μg of protein was loaded per lane. Detection of fluorophores on the Odyssey (Licor) using fluorescent antibodies or on film with horse-radish peroxidase-labeled secondary antibodies was performed. Nuclear extracts were prepared as follows: soluble cytoplasmic extracts were prepared in a buffer containing 20 mM HEPES at pH 7.9, 10 mM KCl, 1 mM EDTA, 0.2 % NP-40, 10 % glycerol with protease, and phosphatase inhibitors followed by centrifugation (15,000×*g*). The nuclear pellet was resuspended in a buffer containing 420 mM NaCl, 20 % glycerol, 20 mM HEPES at pH 7.9, 10 mM KCl, 1 mM EDTA with protease, and phosphatase inhibitors for 30 min 4°C before centrifugation (15000×*g*). For western analysis, 15 μg of soluble nuclear extract was loaded per lane and either actin (Huang et al. 2005; Shitashige et al. 2007; Dai et al. 2009) or histone H3 were used as loading controls.

### RNA extractions and reverse transcription.

Total RNA was then extracted using Qiagen (Toronto, ON, Caanda) RNA Easy kit. The first strand cDNA was obtained by reverse transcription of 20 μg RNA using Superscript II reverse transcriptase (Invitrogen), according to manufacturer’s protocol.

### PCR reactions.

PCR was carried out in ×1 PCR buffer (200 mM Tris-HCl and 500 mM KCl) plus 3 mM MgCl, 200 nM primers, and 200 μM dNTPs as previously described (Perotti et al. 2009).

Amplification of the SMADs were as follows using a SMAD1 forward primer: 5′CGCTATGAATGTGACCAGCTT3′, SMAD5 forward primer: 5′GAGTGGAGAGTCCAGTCTTACCT3′, SMAD8 forward primer: 5′GTCACAGAGTATACACCAAGAAG3′ in the above conditions for 28 cycles at 55°C.

### shRNA preparation, transfection, and transduction.

Four different sequences of shRNA were prepared that targeted different regions of the *Bmpr1a* mRNA (accession number NM_009758). Sequences for shRNA starting at nucleotide positions 1258, 823, and 348, of the BMPR1A gene were obtained from Oligoengine. RNAi explorer from Gene Link was used to create a shRNA starting at sequence 1124 and lamin sequence design. The siRNA Wizard (InvivoGen) was used to create a scrambled sequence of the target identified from the 1124 start position (shScr). The shRNA sequences were cloned into pSuper.retro vector (Oligoengine) using HindIII sites. HC11 cells were transfected with the shRNA containing constructs or with the empty vector (EV) using Lipofectamine 2000 (Invitrogen), according to manufacturer’s protocols and cells were selected using Geneticin resistance to create several different clones of six cell lines (shBMPR1A-1258, shBMPR1A-1124, shBMPR1A-823, shBMPR1A-348, shEV, shLam, and shScr). Alternatively, cells were transfected using polyethylenimine (PEI). A 1-mg/ml solution of linear PEI MW25K (Polyscience Inc. cat. no. 23966, Woodbridge, ON, Canada) was prepared in water and neutralized with HCL. For each well of a six-well plate with cells at 70–80 % confluence, 2 μg DNA and 8 μl PEI were added to 200 μl serum-free RPMI and mixed by vortexing. PEI/DNA/medium mixture was added to cells after 10–15 min incubation for a total of 2 ml of medium. Geneticin treatment of 500–700 μg/ml started after 24 h with fresh media and continued for 10 d. Alternatively, the vectors were transfected into a 293T-based packaging cell line, Phi-NX, (developed by Dr. Garry Nolan, Stanford University) and the HC11 cells transduced with viral particles according to the pSuper.retro instructions.

**Table:** 

	shRNA description	Target sequence
1	shBMPR1A-1258	5′-ACCATTTCCAGCCCTACAT-3′
2	shBMPR1A-1124	5′-GTTGCTGTATTGCTGACCT-3′
3	shBMPR1A-823	5′-AGAAATCTACCAGACGGT-3′
4	shBMPR1A-348	5′-AGCTACGCAGGACAATAG-3′
5	shLam	5′-TGAGGATGACGACGAGGA-3′

## Results

### The BMPR1A pathway is only active in undifferentiated and competent cells.

BMPs are members of the TGF-β superfamily. Activated receptors phosphorylate the DNA-binding proteins SMAD1/5/8, each of which can then heterodimerize with SMAD4 and translocate to the nucleus to regulate transcription of downstream target genes (Itoh et al. 2000). As the activity and function of the BMPR1A pathway in mammary epithelial cells is relatively uncharacterized, we investigated the phosphorylation of SMAD1/5/8 as a read-out of the canonical BMPR1A pathway in the presence of serum.

**Figure 1.**
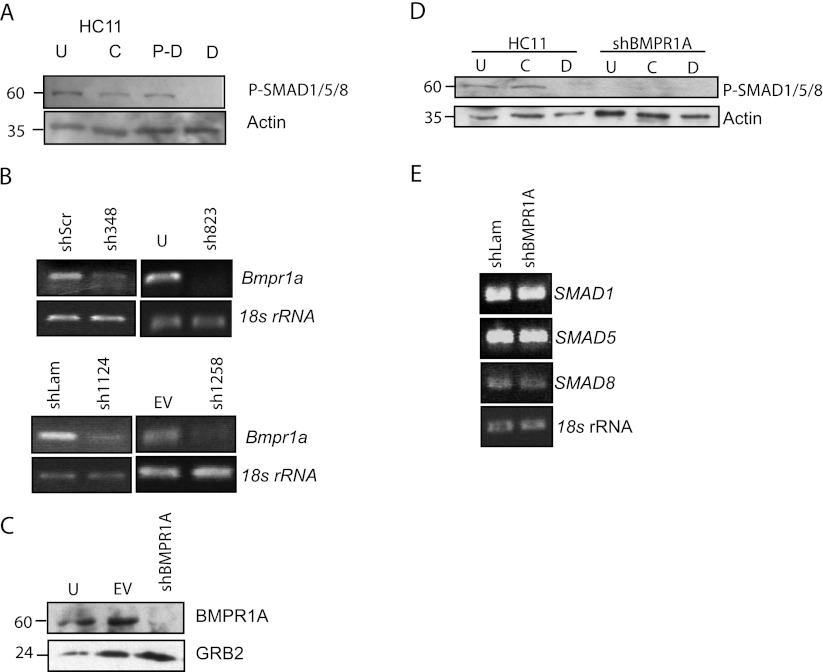
ShRNA directed against *Bmpr1a* reduces mRNA, protein, and SMAD activation. *A*, Western analysis showing phosphorylated SMAD1/5/8 (60 kDa), in nuclear extracts of undifferentiated (*U*), competent (*C*), and pre-differentiated (*P-D*) cells but reduced in differentiated HC11 cells (*D*). Actin is used as a loading control. *B*, The expression of *Bmpr1a* was analyzed by PCR in four knock-down cell lines, shBMPR1A-348, shBMPR1A-823, shBMPR1A-1124, and shBMPR1A-1258, and compared with expression in control cell lines, shScr, undifferentiated HC11, shLam, or empty vector (*EV*) cells. Amplification of the gene encoding 18S rRNA was used as a loading control. *C*, Analysis of BMPR1A protein (59 kDa) in shBMPR1A-1258 by western using the loading control (GRB2, 24 kDa). *D*, Absence of phosphorylated SMAD1/5/8 at all stages, in nuclear extracts of HC11 cells, stably expressing shBMPR1A-1258. *E* Expression of SMAD1/5/8 in undifferentiated cells.

The activation of the BMPR1A pathway in parental and shBMPR1A-stable cell lines was assessed using an antibody directed against phosphorylated-SMAD1/5/8. We determined that the pathway is active in undifferentiated (treated with EGF and insulin), competent (treated with EGF), and pre-differentiated (treated with dexamethasone and insulin) parental HC11 cells but that the activity is greatly reduced in the differentiated cells, which are those treated additionally with prolactin (Fig. 1*A*).

### Knockdown of Bmpr1a with short-hairpin RNA.

In order to characterize the function of BMPR1A, we knocked down *Bmpr1a* expression in HC11 cells. We separately transfected four different vector constructs, each encoding a different short hairpin (shRNA) against *Bmpr1a* (shBMPR1A) that targeted different regions of the gene, creating a minimum of four separate stable HC11-shBMPR1A cell lines (shBMPR1A-348, shBMPR1A-823, shBMPR1A-1124, and shBMPR1A-1258), one with shRNA directed against lamin A (shLam), scrambled-sh1124 (shScr) and one empty vector line (shEV). The four distinct shBMPR1A sequences and six cell lines were generated in order to greatly reduce the chance of off-target effects. In addition, we made several distinct stable cell line clones with each of the constructs as experimental replicates. Overall, the results in the study were consistent across the replicates and each of the different shBMPR1A sequences, providing multiple levels of experimental replication.

*Bmpr1a* gene expression was analyzed by PCR before and after knockdown. Compared with the control cells, HC11 cells expressing one of the four shBMPR1A constructs had greatly reduced amounts of *Bmpr1a* mRNA (representative lines, Fig. 1*B*). HC11 cells expressing an shRNA directed against the unrelated target gene, lamin, did not have reduced *Bmpr1a* expression (Fig. 1*B*), providing an indication of specificity of the BMPR1A shRNA. The level of knockdown in each stable cell line was confirmed prior to performing additional experiments.

The effect of knockdown on BMPR1A protein levels was also investigated using western blot and portrayed with one representative shRNA sequence, shBMPR1A-1258 (Fig. 1*C*). The relative level of BMPR1A protein in shBMPR1A-1258, was considerably reduced compared with the undifferentiated parental cell line or empty vector control line.

The reduction of BMPR1A using shRNA (shBMPR1A-1258 shown as representative of all lines) resulted in loss of SMAD1/5/8 phosphorylation (Fig. 1*D*), indicating that this pathway is no longer active and thus not functioning in either the undifferentiated or competent cells, in addition to the differentiated cells that already had reduced BMPR1A pathway activation as compared with the parental cell line or one carrying the empty vector. This was consistent among all four shRNA sequences directed against *Bmpr1a*. Although the phosphorylation of individual SMADs was not tested, SMAD1, SMAD5 and SMAD8 are each expressed in undifferentiated HC11 cells and their gene expression unaffected by knockdown of BMPR1A (Fig. 1*E*). The reduction in BMPR1A mRNA and protein by shRNA therefore results in the abrogation of endogenous canonical SMAD1/5/8 phosphorylation.

### BMPR1A is required for mammary epithelial cell differentiation.

The BMP/BMPR1A signalling pathway can influence cell behaviour in many different ways, and BMPs have been identified as important cell fate regulators, required for the differentiation of other epithelial cells such as hair follicle cells.

**Figure 2.**
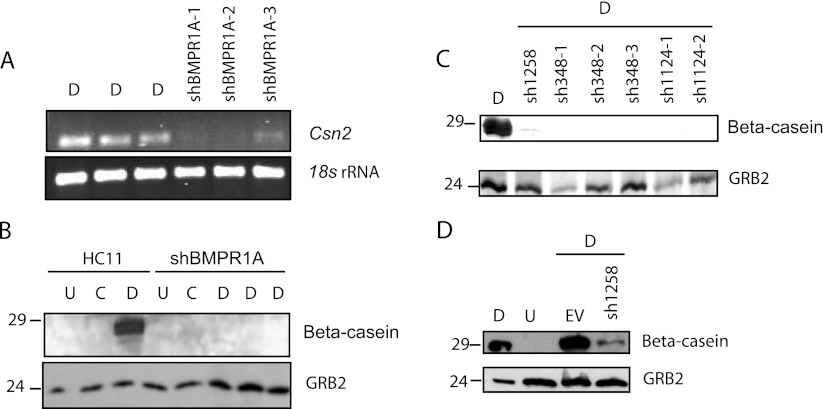
BMPR1A is required prior to lactogenic signaling for the production of beta-casein. *A*, The PCR amplification of beta-casein (*Csn2*) from cDNA prepared from fully differentiated HC11 cells was compared with shRNA expressing cells that were also similarly treated with lactogenic hormones. Three independent cell lines of a representative shBMPR1A, sh1258, are shown. Amplification of 18s rRNA was used as loading control. *B*, The presence of beta-casein (29 kDa) was analyzed by western blot. Beta-casein production was assessed in parental HC11 cells across three stages of differentiation as a positive control and in shBMPR1A-HC11 cells treated similarly with lactogenic hormones, with three independent replicates of cell lines carrying shBMPR1A-1258. *C*, Western analysis, using the Odyssey (fluorescent secondary antibody), of beta-casein production in fully differentiated HC11 cells of the parental HC11 line was compared with different shBMPR1A lines similarly treated. Each *lane*, represents independent experiments. Overexposure reveals a faint signal in *lane 2*. *D*, Western analysis of undifferentiated and differentiated HC11 cells or cells stably expressing the empty vector (*EV*) or shBMPR1A-1258 treated with lactogenic hormones. Overexposure reveals a faint signal in *lane 4*.

To determine if the reduction in BMPR1A by shRNA affected mammary epithelial differentiation, total RNA and soluble proteins were extracted from each of the differentiation stages of the BMPR1A knockdown cell lines together with the control HC11 cell lines and we investigated the expression of the terminal alveolar cell differentiation marker, beta-casein. Using PCR analysis, it is apparent that there is little to no beta-casein (*Csn2*) gene expression in three independent clones of the representative shBMPR1A sequence, shBMPR1A-1258, that were treated with lactogenic hormones, in contrast to the parental cell lines (Fig. 2*A*).

Additionally, there is little to no production of beta-casein protein in the shBMPR1A-1258 clones (Fig. 2*B*) or in any of the shBMPR1A cell lines and clones upon treatment with lactogenic hormones (Fig. 2*C*). Multiple stable cell lines carrying the same shRNA and as well, multiple cell lines each with different shRNA sequences all directed against *Bmpr1a* expression produced similar results. As a negative control, the level of beta-casein was unaffected by stable presence of the empty vector as compared with parental or the shBMPR1A-1258 cells (Fig. 2*D*). These results indicate the requirement of BMPR1A and SMAD1/5/8 activation, prior to differentiation, for the ultimate differentiation of mammary epithelial cells into milk-secreting alveolar cells.

### Noggin antagonizes mammary epithelial cell differentiation.

**Figure 3.**
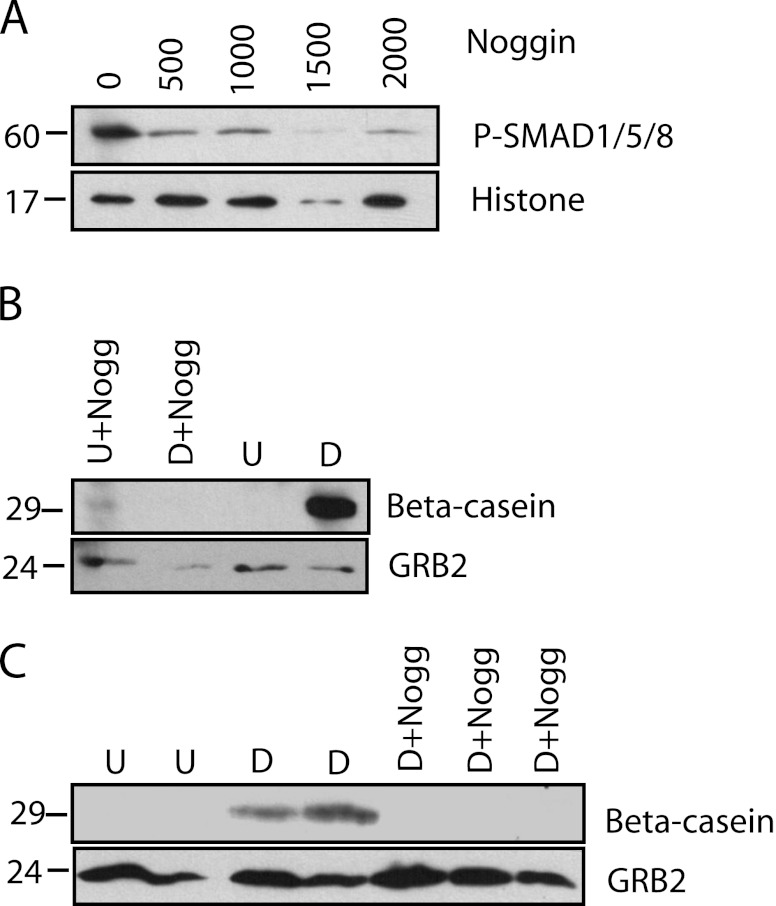
The BMP antagonist, Noggin, reduces SMAD1/5/8 phosphorylation and abrogates beta-casein production. *A*, Western analysis of SMAD1/5/8 phosphorylation from nuclear extracts in the presence of increasing doses of Noggin (in nanograms per milliliter). Histone H3 is a loading control. *B*, Western analysis of beta-casein production in HC11 cells cultured in the presence or absence of Noggin (2,000 ng/ml) in their undifferentiated through to their differentiated state. *C*, Westerns for beta-casein production in experimental replicates of HC11 cells treated or not with Noggin from their undifferentiated to differentiated state. Undifferentiated (*U*), competent (*C*), differentiated HC11 cells (*D*), and Noggin (*Nogg*).

To validate our observations, we also tested the effect of the BMP antagonist, Noggin, on HC11 cell differentiation. Noggin antagonizes BMP-2, BMP-4, and BMP-7, preventing their interaction with the receptor (for review, see Botchkarev 2003). Treatment of undifferentiated cells with increasing doses of Noggin results in a severe decrease of phospho-SMAD1/5/8 (Fig. 3*A*). When these cells were induced to differentiate, there was no beta-casein production compared with the control cells (Fig. 3*B*, *C*). Together, this indicates that the BMP-BMPR1A pathway is important for the phase of mammary epithelial cell differentiation characterized by beta-casein production.

## Discussion

We determined that in mouse HC11 cells, differentiation into milk-producing cells was blocked with the loss the BMPR1A pathway prior to differentiation. Our investigation into the function of BMPR1A in the differentiation of mammary epithelial cells is the first indication that it could function later in mammary gland development in addition to its role in embryonic mammary gland formation.

The present analysis was based upon our previous screen to identify differentially expressed genes upon lactogenic hormone stimulation that could potentially have a role in regulating differentiation (Perotti et al. 2009). The gene for BMPR1A was preferentially expressed in undifferentiated cells, although the BMPR1A protein was present at all stages of HC11 differentiation. In this report, we determined that the BMPR1A-SMAD1/5/8 pathway is active in undifferentiated and pre-differentiated cells, but not, or is greatly reduced, in differentiated cells. It was previously reported that the activity of the estrogen receptor differs during HC11 differentiation although the estrogen receptor is expressed at all phases, potentially reflecting corepressor levels (Faulds et al. 2004). Therefore differential regulation of signal transduction pathways is possible across HC11 differentiation.

We demonstrated that the BMPR1A-SMAD1/5/8 pathway is greatly reduced upon treatment with prolactin. Prolactin is essential for lobuloalveolar differentiation (Horseman et al. 1997; Brisken et al. 1999) and may negatively affect the upstream BMPR1A-SMAD1/5/8 pathway. Prolactin inhibition of upstream pathways has been previously reported for cross-talk of the prolactin pathway with that of EGF (Haines et al. 2009). Disruption of BMPR1A expression prior to differentiation, in undifferentiated mammary epithelial cells and/or competence phases where extracellular matrix proteins are produced (Chammas et al. 1994), prevented cellular differentiation and beta-casein production. This demonstrates a requirement of the BMPR1A receptor for differentiation that is upstream of lactogenic signaling, potentially that of insulin, dexamethasone and/or prolactin. BMPR1A is required for the functional differentiation of mammary epithelial cells into milk-producing cells. Additional studies are required to explore the intricate cross-talk regulating these pathways.

Previous studies examined the phenotype of the conditional Keratin-14-Cre-BMPR1A null mouse, which targeted expression in the epithelial cells. The conditional nulls had normal mammary glands at the embryonic stage (Andl et al. 2004), indicating that the deletion of BMPR1A in Keratin-14 expressing cells has little effect on early mammary gland development. The effects of conditional deletion of BMPR1A at later stages of mammary gland development were not examined. Keratin-14 expression is essentially restricted to the basal cell layer of the mammary epithelium. It is possible, however, that BMR1A functions in the luminal cell compartment to permit alveolar cell differentiation. Our results are consistent with what is known about the role of BMPR1A in other epithelial cells, such as hair follicles. In mice, BMPR1A is required for differentiation of the inner root sheath (Andl et al. 2004) and hair shaft (Kobielak et al. 2003; Andl et al. 2004).

Transgenic doxycycline-induced expression of a conditional dominant-negative BMPR2 receptor in the mammary carcinoma model, mouse mammary tumor virus polyoma middle T antigen, did not result in disruption of mammary gland formation, at least in adult mice. The glands were not examined during pregnancy, and the endogenous BMPR2 receptors were still expressed in this mouse system. The study did indicate a tumor-suppressive role for BMPR2 (Owens et al. 2012).

Recent studies have demonstrated that mutational inactivation of BMP signaling pathways is critical to the pathogenesis of sporadic and inherited human cancer. Inherited inactivating mutations of both the Bmpr1-a/Alk3 type I receptor and the SMAD4 signaling molecule cause familial juvenile polyposis, an inherited gastrointestinal cancer predisposition syndrome (Howe et al. 1998; Howe et al. 2001) (Zhou et al. 2001; Waite and Eng 2003). There is currently little knowledge about BMP ligand and receptor function in breast cancer. BMP6 has been detected in some breast cancer cells and primary tumors (Clement et al. 1999) and BMP7 has been reported to be present in different primary breast cancers (Schwalbe et al. 2003). The mRNA levels of BMP receptor and ligand are greater in cells with high metastasic potential (Arnold et al. 1999). However, BMP2 has been shown to inhibit proliferation in breast cancer cell lines that express SMAD1 and SMAD4 (Ghosh-Choudhury et al. 2000; Pouliot and Labrie 2002). BMP2 has also been shown to enhance the migration of MCF7 breast cancer cell line and its over-expression promotes tumor formation in a breast cancer xenograft model (Clement et al. 2005). Clearly, more research is required to understand the role of BMPR1A in the normal and diseased mammary gland.

## Conclusions

Our results suggest that the BMP-BMPR1A pathway, possibly via SMAD1/5/8, is required in undifferentiated and/or competent HC11 cells to allow the proper differentiation of mammary milk-secretory cells. This is the first analysis of BMPR1A function in mammary epithelial cell differentiation, and our results indicate that the pathway is required before lactogenic hormone signalling for the production of beta-casein.
